# Diabetic nephropathy in a sibling and albuminuria predict early GFR decline: a prospective cohort study

**DOI:** 10.1186/1471-2369-14-124

**Published:** 2013-06-17

**Authors:** Douglas Gunzler, Anthony J Bleyer, Robert L Thomas, Alicia O’Brien, Gregory B Russell, Abdus Sattar, Sudha K Iyengar, Charles Thomas, John R Sedor, Jeffrey R Schelling

**Affiliations:** 1 Department of Medicine, Case Western Reserve University, 2500 MetroHealth Drive, Rammelkamp R415, Cleveland, OH, 44109, USA; 2 Department of Medicine, Division on Nephrology, Wake Forest University, Medical Center Blvd, Winston Salem, NC, 27157, USA; 3 Department of Epidemiology and Biostatistics, Case Western Reserve University, 2500 MetroHealth Drive, Rammelkamp R415, Cleveland, OH, 44109, USA; 4 Department of Physiology and Biophysics, Case Western Reserve University, 2500 MetroHealth Drive, Rammelkamp R415, Cleveland, OH, 44109, USA

**Keywords:** Albuminuria, CKD, Diabetes, ESRD, Genetics, Progression, Proteinuria

## Abstract

**Background:**

Diabetic nephropathy is a growing clinical problem, and the cause for >40% of incident ESRD cases. Unfortunately, few modifiable risk factors are known. The objective is to examine if albuminuria and history of diabetic nephropathy (DN) in a sibling are associated with early DN progression or mortality.

**Methods:**

In this longitudinal study of adults >18 yrs with diabetes monitored for up to 9 yrs (mean 4.6 ± 1.7 yrs), 435 subjects at high risk (DN family history) and 400 at low risk (diabetes >10 yrs, normoalbuminuria, no DN family history) for DN progression were evaluated for rate of eGFR change using the linear mixed effects model and progression to ESRD. All-cause mortality was evaluated by Kaplan-Meier analyses while controlling for baseline covariates in a Cox proportional hazards model. Covariates included baseline eGFR, age, gender, race, diabetes duration, blood pressure, hemoglobin A1c and urine albumin:creatinine ratio. Propensity score matching was used to identify high and low risk group pairs with balanced covariates. Sensitivity analyses were employed to test for residual confounding.

**Results:**

Mean baseline eGFR was 74 ml/min/1.73 m^2^ (86% of cohort >60 ml/min/1.73 m^2^). Thirty high risk and no low risk subjects developed ESRD. eGFR decline was significantly greater in high compared to low risk subjects. After controlling for confounders, change in eGFR remained significantly different between groups, suggesting that DN family history independently regulates GFR progression. Mortality was also significantly greater in high versus low risk subjects, but after controlling for baseline covariates, no significant difference was observed between groups, indicating that factors other than DN family history more strongly affect mortality. Analyses of the matched pairs confirmed change in eGFR and mortality findings. Sensitivity analyses demonstrated that the eGFR results were not due to residual confounding by unmeasured covariates of a moderate effect size in the propensity matching.

**Conclusions:**

Diabetic subjects with albuminuria and family history of DN are vulnerable for early GFR decline, whereas subjects with diabetes for longer than 10 years, normoalbuminuria and negative family history, experience slower eGFR decline, and are extremely unlikely to require dialysis. Although we would not recommend that patients with low risk characteristics be neglected, scarce resources would be more sensibly devoted to vulnerable patients, such as the high risk cases in our study, and preferably prior to the onset of albuminuria or GFR decline.

## Background

Diabetes is a burgeoning public health problem, with an estimated prevalence of 25.8 M children and adults in the U.S. (http://www.diabetes.org/diabetes-basics/diabetes-statistics/). Diabetic nephropathy (DN) is one of the most feared complications, and the etiology for over 40% of incident end stage renal disease (ESRD) cases in the U.S. Since only a minority of diabetic patients develop DN, it is important to identify the at-risk population, prior to GFR decline.

Hyperfiltration is the earliest measurable biomarker for DN progression in types 1 and 2 diabetes. However in type 2 diabetes, which comprises the vast majority of DN, the mean duration between disease onset and diagnosis commonly exceeds the window for hyperfiltration detection [1]. Microalbuminuria is a practical alternative, but its reliability as a predictor of DN progression has been questioned, due to frequent reversion to normoalbuminuria [2], and the occurrence of typical DN histopathology in the absence of albuminuria [3,4]. Intensive effort is therefore being invested to identify more reliable biomarkers.

Microalbuminuria is, however, an accepted risk factor for all-cause and cardiovascular mortality [5]. Since estimated GFR (eGFR) and the rate of eGFR decline are also associated with mortality [6-10], staging systems, which combine proteinuria and eGFR, have been proposed for identification of the highest risk population for death and ESRD [10,11]. Although these strategies provide an efficient mechanism for screening large populations, they tend to focus on advanced disease, which may not be as amenable to therapies.

Because DN is heritable [12], we have advocated that diabetic siblings should be vigilantly targeted for reduction of modifiable risks for DN progression, such as blood pressure and glycemia [13]. Early therapeutic intervention, before GFR and albuminuria are grossly abnormal, is desirable, but whether DN family history is a discriminator in this subpopulation is unclear. To examine the hypothesis that DN family history and albuminuria predict early DN progression, we have assembled a longitudinal cohort of diabetic subjects, with relatively preserved baseline GFR, who were evaluated for rate of eGFR change, progression to ESRD, and mortality, after adjusting for relevant covariates.

## Methods

### Study participants

The study design has been described [13]. Briefly, high risk subjects were defined as diabetic siblings to patients with ESRD due to DN (i.e., DN family history). A distinction between type 1 and 2 diabetes was not made, though based upon mean age at diabetes diagnosis >40 years, the cohort is presumed to be almost entirely type 2. Exclusion criteria included eGFR <20 mL/min/1.73 m^2^, chronic dialysis or renal transplantation. Low risk subjects were defined by diabetes >10 years, urine albumin:creatinine ratio <30 mg/g at study entry, and absence of ESRD family history in first or second degree relatives.

Participants were enrolled beginning April 1, 2003, monitored annually for eGFR through June 30, 2011, and for mortality through March 31, 2012. Annual evaluations included a standardized questionnaire for demographic and medical information. Sitting BP, measured at the brachial artery with a manual sphygmomanometer, was recorded by research nurses.

The study protocol was approved by the Institutional Review Boards at Case Western Reserve University affiliated hospitals (MetroHealth System, University Hospitals Case Medical Center, Louis Stokes Cleveland VA Medical Center) and Wake Forest University, and all subjects provided written consent for enrollment.

### Serum creatinine assay

Serum creatinine was measured as described [13]. Beginning May 20, 2007, creatinine assays used isotope dilution mass spectrometry (IDMS)-traceable reagent methods. Forty serum specimens spanning the range of creatinine concentrations were re-assayed with the Cleveland Clinic Laboratory reference standard assay to assess precision (Additional file 1: Figure S1).

### Cystatin C assay

Serum cystatin C was measured from stored frozen samples by a particle-enhanced immunonephelometric assay (N Latex Cystatin C, Siemens Healthcare Diagnostics, Deerfield, IL) with a BNII nephelometer (Siemens) at the University of Minnesota. To avoid assay drift over time, all samples were assayed within a 30-day period.

### Albumin assay

Urine albumin concentration was initially determined by radioimmunoassay as described [13]. This assay was discontinued in 2007, and thereafter urine albumin was assayed by a turbidometric method with a Beckman Coulter LX20 analyzer. Assays on 20 randomly selected samples by both methods revealed that no calibration correction was required.

### Urine creatinine assay

Urine creatinine concentration was determined as described [13].

### Hemoglobin A1c (HbA1c) assay

HbA1c assays were performed as described [13].

### Estimated glomerular filtration rate (eGFR) calculations

eGFR based on serum creatinine (eGFR_creat_) was calculated from the MDRD Study equations: eGFR_creat_ (mL/min/1.73 m^2^) = 186 × creatinine (mg/dL)^−1.154^ × age^−0.203^ × 0.742 (if female) × 1.212 (if African American) prior to May 20, 2007 [14], or eGFR_creat_ (mL/min/1.73 m^2^) = 175 × creatinine (mg/dL)^−1.154^ × age^−0.203^ × 0.742 (if female) × 1.212 (if African American) after May 20, 2007 [14]. For eGFR calculations based upon serum creatinine plus cystatin C values (both in mg/dl), we used the modified CKD-EPI equation, eGFR_creat+cysC_ (mL/min/1.73 m^2^) = 177.6 × creatinine^-0.65^ × [-0.105 + (1.13 x CysC)]^-0.57^ × age^-0.20^ × (0.82 if female) × (1.11 if black) [14]. For calculations based upon standardized cystatin C only, we used the CKD-EPI equation eGFR_cysC_ (mL/min/1.73 m^2^) = 127.7 × [-0.105 + (1.13 × CysC)]^-1.17^ × age^-0.13^ × (0.91 if female) × (1.06 if black) [14].

Since samples assayed for cystatin C assays represented a subset (56%) of the samples assayed for creatinine, to account for missing values, we imputed plausible eGFR_cysC_ and eGFR_creat+cysC_ values to match eGFR_creat_ observations using PROC MI in SAS Version 9.2. Multiple imputations were performed, creating five data sets for valid inference using the Markov Chain Monte Carlo algorithm [15]. After running separate mixed effects models for each imputed data set, we used PROC MIANALYZE to combine the results.

### Statistical analyses for risks of DN progression

We compared risk group differences in baseline characteristics, eGFR and mortality via chi-square, t-tests and Wilcoxon rank-sum tests. The linear mixed effects model [16] was used to evaluate the mean change in eGFR (by all equations) between high and low risk subjects. This model is an extension of linear regression for longitudinal data, and is specifically designed to handle correlated repeated measurements, missing data and dropouts. The baseline covariates that were adjusted in our models included age, gender, race, systolic BP, diastolic BP, HbA1c, diabetes duration, eGFR, and urine albumin:creatinine ratio. Longitudinal models including these baseline covariates assessed whether eGFR change is: (1) different in the high and low risk groups (risk group by time interaction effect), (2) constant over time [time effect (years)], and (3) different between risk groups under the assumptions that the eGFR values differ (i.e., at baseline) and progression curves remain parallel (group main effect), while controlling for covariates. The interaction between risk group and time, depicted by β, in the unadjusted models describes eGFR slope differences in high vs. low risk groups. In the covariate model, the interaction, depicted by β_Basic_, adjusts for baseline covariates in order to describe the impact of family history on eGFR change. Propensity score techniques were used to identify matched sets of patients from the high and low risk groups with similar baseline covariate distributions [17,18]. We used a logistic regression model to estimate propensity to be a high risk subject based on all baseline covariates: age, gender, race, diabetes duration, systolic BP, diastolic BP, HbA1c, eGFR_creat_, and urine albumin:creatinine ratio. Sensitivity analysis was then used to determine the impact of residual confounding in the matched pair subsample [18].

As an alternative renal outcome to eGFR decline, we also compared the number of high versus low risk subjects that progressed to ESRD. A separate analysis was also undertaken to assess the effect of albuminuria on eGFR decline, using the linear mixed effects model. This analysis was conducted in high risk subjects only, because low risk subjects required normoalbuminuria upon enrollment. The effects of 30 mg/g (microalbuminuria) and 300 mg/g macroalbuminuria) thresholds were tested in the model.

### Statistical analyses for mortality

Every study subject was evaluated for date of death by a Social Security Death Index database search (http://www.genealogybank.com/gbnk/ssdi/). Mortality data were analyzed to compare time until death between risk groups using a Kaplan-Meier plot and log-rank test for unadjusted analyses. A Cox proportional hazards model also included the baseline covariates age, gender, race, systolic BP, diastolic BP, HbA1c, diabetes duration, baseline eGFR, and urine albumin:creatinine ratio to examine the effect of DN family history on risk of death [19]. Eighty-seven per cent of subjects in the low risk group and 74% of subjects in the high risk group were right censored.

We also ran an extended Cox regression model to estimate the hazard risk, including all the covariates, but the baseline measurements were replaced with the annual measurements for systolic BP, diastolic BP, HbA1c and urine albumin:creatinine ratios. This analysis used data from the first seven repeated measures, as additional data were too sparse (n = 16 for each of the four time-varying covariates at the eighth repeated measure). We used the counting process style of input in PROC PHREG in SAS Version 9.2 to handle the time-varying covariates in the extended Cox regression model [20].

Finally, a Cox proportional hazards model was applied to the high and low risk groups matched for baseline covariates, which were then stratified by the matched pairs, to evaluate if risk group had an effect on mortality after reducing the impact of selection.

## Results

### Patient characteristics

Diabetic subjects were segregated according to high and low risk for DN as defined in Methods. Baseline characteristics are shown in Table 1. Of note, eGFR_creat_ = 74 ml/min/1.73 m^2^ for the cohort, and 86% of subjects had eGFR >60 ml/min/1.73 m^2^ at study inception, indicating that progression rates were calculated from a preserved baseline GFR in most instances. Of factors that differed significantly between risk groups, diabetes duration and albuminuria were anticipated, based upon risk group definitions. Use of lipid-lowering agents (predominantly statins) was also greater in the low risk group. Baseline information for each group was calculated according to recruitment center (Case Western Reserve University, Wake Forest University). Because few between-center differences were observed (Additional file 1: Table S1), data are combined in all subsequent analyses.

**Table 1:** Baseline characteristics by risk group

	**High risk**	**Low risk**	**p-value**
**N**	435	400	
**Gender (% female)**	62.8	67.0	0.20
**Age (yr)**	58.9 ± 10.5	59.9 ± 11.8	0.20
**Race (% AA)**	51.4	52.5	0.75
**Diabetes duration (yr)**	14.1 ± 9.7	17.2 ± 8.7	< 0.01
**HbA1c (%)**	7.7 ± 1.9	7.8 ± 2.0	0.46
**Systolic BP (mm Hg)**	135.4 ± 17.8	134.2 ± 19.7	0.36
**Diastolic BP (mm Hg)**	75.9 ± 11.6	74.7 ± 11.9	0.14
**Serum creatinine (mg/dl)**	1.05 ± 0.37	0.97 ± 0.25	<0.01
**eGFR**_**creat **_**(ml/min/1.73 m**^**2**^**)**	74.3 ± 27.7	76.1 ± 24.3	0.32
**Serum cystatin C (mg/dl)**	1.1 ± 0.3	1.0 ± 0.5	<0.01
**eGFR**_**creat+cysC **_**(ml/min/1.73 m**^**2**^**)**	73.9 ± 29.3	77.4 ± 24.1	0.06
**Urine alb:creat (mg/g)**	27.6 (9-119)	11.2 (6-22)	<0.01
**BP meds (%)**	77.7	81.6	0.16
**ACE inhibitor (%)**	48.5	52.8	0.21
**ARB (%)**	14.1	14.8	0.77
**Other BP meds (%)**	57.3	57.4	0.98
**Insulin (%)**	45.8	42.9	0.26
**Oral hypoglycemic agents (%)**	61.0	65.6	0.06
**Lipid-lowering agents (%)**	64.2	70.2	0.01

Data are presented as mean ± standard deviation for continuous measures, % frequency of reference level for discrete measures and median (first quartile-third quartile) for urine alb:creat (mg/g). Analyses to generate p-values include t-tests, χ^2^ tests and Wilcoxon rank-sum tests where appropriate. Abbreviations: ACE, angiotensin converting enzyme; alb:creat, albumin to creatinine ratio; ARB, angiotensin receptor blocker; BP, blood pressure; GFR, glomerular filtration rate; HbA1c, hemoglobin A1c.

### Risks for DN progression

**Figure 1:**
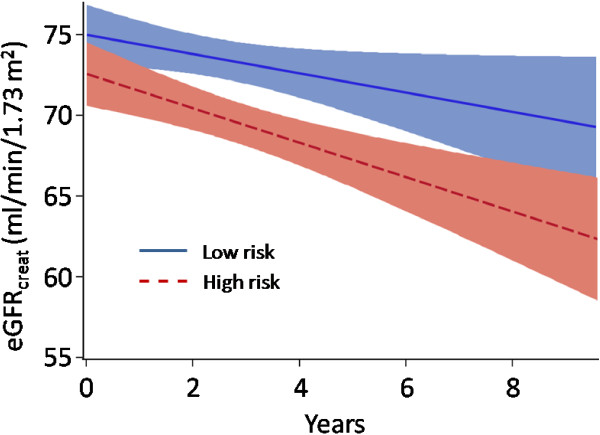
**Unadjusted eGFR change over time.** Linear regression of eGFR_creat_ was plotted against time. Shaded areas represent 95% confidence intervals. The two regression lines are significantly different (p <0.001).

Strikingly, 30 high risk and no low risk subjects developed ESRD. The unadjusted, annualized eGFR_creat_ change was significantly different between risk groups (Figure 1, β = -1.65, p <0.001). The difference in eGFR_creat+cysC_ (β = -0.97, p = 0.10) and eGFR_cysC_ (β = -0.62, p = 0.40) change between risk groups was not statistically significant (not shown). Equations to describe mean change of eGFR_creat_ also demonstrate greater decline in high risk [eGFR_creat_ = (-2.19 × years) + 73.29, in ml/min/1.73 m^2^], compared to low risk [eGFR_creat_ = (-0.78 × years) + 75.16, in ml/min/1.73 m^2^] subjects.

**Figure 2:**
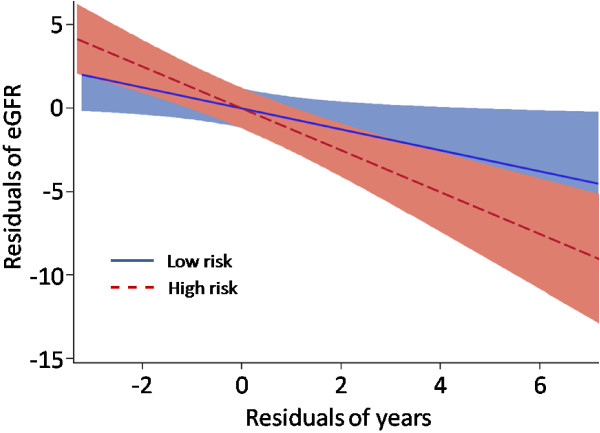
**Partial regression plot of eGFR progression.** A linear regression model approach was used to generate a partial regression plot for eGFR_creat_, adjusting for covariates shown in Table 2. Shaded areas represent 95% confidence intervals. The plot is derived from several different regression equations. Initially, the residual errors of a regression equation were obtained, with (1) eGFR as the outcome, and all covariates besides years, as the independent variables, and (2) years as the outcome against all other covariates as the independent variables. These two steps were conducted separately for the high and low risk groups, and the resulting residual errors were then plotted against each other. Note that values on both axes are residuals, rather than actual eGFR_creat_ values. Furthermore, because analyses were conducted separately for high and low risk groups, by definition, both lines intersect at the graph origin (0,0). The high and low risk groups are significantly different (β_Basic_ = -1.74, p <0.001).

A significant association was observed between eGFR_creat_ change and baseline eGFR_creat_, albuminuria, HbA1c, systolic and diastolic blood pressure in a mixed effects model (Table 2). As an alternative means of assessing a difference in eGFR between high and low risk groups, a partial regression plot was constructed, whereby the plot displayed the relationship of eGFR against time, after adjusting for the significant baseline covariates. Figure 2 reveals that eGFR_creat_ decline remained significantly greater in the high risk group (β_Basic_ = -1.74, p <0.001). An analogous model for eGFR_creat+cysC_ showed significant associations with baseline eGFR_creat+cysC_ and albuminuria only (Additional file 1: Table S2), and following adjustment eGFR_creat+cysC_ decline was still significantly greater for high risk subjects (β_Basic_ = -1.38, p = 0.001). After accounting for covariates, the remaining variable that distinguishes the high and low risk groups is family history of DN. Therefore, persistent differences in rate of eGFR decline between risk groups, after covariate adjustment, suggest that DN family history is a factor in the progression of DN.

**Table 2:** **Baseline covariate effects on eGFR**_**creat **_**using linear mixed effects model parameter estimates, 95% confidence intervals and p-values**

**Effect**	**Estimate (β**_**Basic**_**)**	**95% CI**	**p-value**
**Risk group (high vs. low)**	1.57	(-1.49, 4.63)	0.313
**Year**	-0.93	(-1.34, -0.52)	<0.001
**Risk group by year interaction**	-1.74	(-2.56, -0.92)	<0.001
**Diabetes duration**	0.02	(-0.08, 0.12)	0.708
**eGFR**_**creat**_	0.73	(0.69, 0.77)	<0.001
**Urine albumin:creatinine ratio**	-4.40	(-6.09, -2.71)	<0.001
**Systolic BP**	-0.08	(-0.14, -0.02)	0.010
**Diastolic BP**	0.13	(0.03, 0.23)	0.010
**HbA1c**	-0.65	(-1.30, 0.00)	0.046

**Figure 3:**
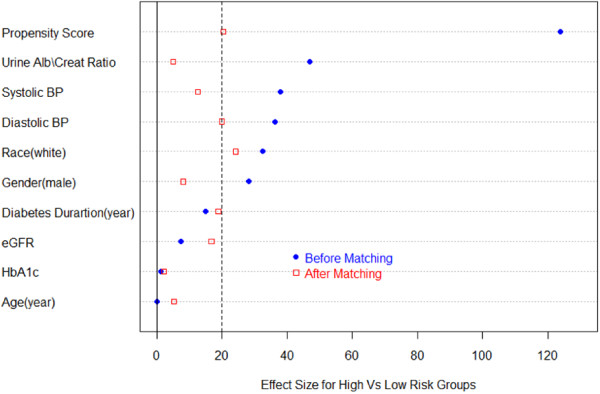
**Plot of effect sizes (difference in means divided by the standard error) comparing propensity to be a high risk subject and covariate differences between risk groups before and after propensity matching.** An effect size ≤ 0.20 (at the dashed line) represents a relatively small difference between risk groups on the propensity score or covariate.

Using a greedy matching algorithm for propensity matching on the propensity scores from the logistic regression model, covariates were more similar between risk groups within the matched pair subsample (Figure 3, N = 199 pairs), thus reducing the impact of selection in the subsample. Using only matched subjects with ≥3 eGFR_creat_ measurements (N = 155) we estimated individual slopes for eGFR_creat_ in a linear regression model, by regressing eGFR_creat_ against year for each individual. Significant differences in individual estimated slopes by risk group for the matched pairs were observed (*p* <0.001, by non-parametric Wilcoxon signed-rank test). These data provide strong evidence for significant eGFR_creat_ decline in the high risk group, and imply that family history of DN is a factor in the progression of DN.

A sensitivity analysis was performed to examine the degree of hidden bias that would be necessary to explain the observed association between eGFR decline and risk group in the 155 matched pair subset. We determined that a hidden bias or unaccounted covariate would need to cause an increase of the slope differences in high vs. low risk groups by more than 50%. Therefore, the association cannot be attributed to hidden biases or unmeasured covariates with only small effects.

### Albuminuria as a biomarker for DN progression

One criterion that distinguishes high and low risk groups is absence of microalbuminuria in the low risk group. To further evaluate the role of albuminuria on GFR decline, we employed a linear mixed effects model, considering both macroalbuminuria (>300 mg/g urine albumin: creatinine ratio) and microalbuminuria (30-299 mg/g) thresholds. Decline in eGFR_creat_ was significantly greater in subjects above the 300 mg/g threshold (p < 0.01), while the 30 mg/g threshold showed a trend toward larger decline (p = 0.054). The correlation between albuminuria and change in eGFR_creat+cysC_ or eGFR_cysC_ was not significant.

### Risks for mortality

**Figure 4:**
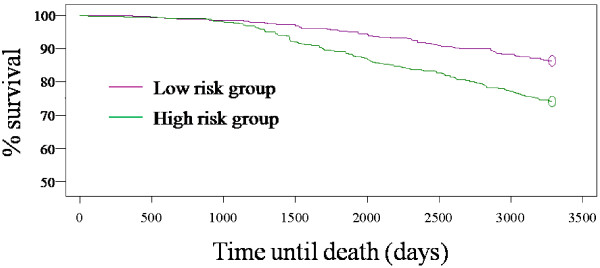
Kaplan–Meier curves for mortality in the high and low risk groups (p <0.001).

Over the duration of the study, 119 high risk and 54 low risk subjects died. The Kaplan-Meier plot of cumulative risk of death in high versus low risk subjects is shown in Figure 4 (Log-rank test χ^2^ = 20.41, p <0.001). There was a trend toward faster DN progression in those who died, as shown by the unadjusted (β = -0.03, p = 0.089) and baseline covariate-adjusted eGFR_creat_ (β_Basic_ = -2.00, p = 0.094) change over time.

However, using a Cox proportional hazards model, after correcting for the confounding effects of albuminuria, baseline eGFR, age, gender, race, diabetes duration, BP, and HbA1c, the difference in time until death between the high and low risk groups was no longer statistically significant (Table 3). The results suggest that the significant covariates, albuminuria, age, and gender (Table 3) exert more pronounced effects, compared to DN family history, upon mortality.

**Table 3:** Effect of risk group and baseline covariates on time until death in a Cox proportional hazards model after adjusting for all covariates

**Effect**	**Hazard ratio**	**95% CI**	**p-value**
**Risk group (high vs. low)**	1.17	(0.73, 1.89)	0.521
**Age**	1.06	(1.04, 1.09)	<.001
**Gender (male vs. female)**	1.99	(1.28, 3.09)	0.002
**Race (non-white vs. white)**	1.52	(0.95, 2.43)	0.078
**Diabetes duration**	1.00	(0.99, 1.02)	0.829
**eGFR **_**creat**_	0.99	(0.99, 1.00)	0.273
**Urine alb:creat ratio**	1.28	(1.10, 1.50)	0.002
**Systolic BP**	1.00	(0.99, 1.01)	0.857
**Diastolic BP**	1.01	(0.99, 1.03)	0.373
**HbA1c**	1.10	(0.98, 1.24)	0.104

After using an extended Cox regression model to correct for the confounding effects of baseline eGFR, age, gender, race, and diabetes duration, and the repeated measures of albuminuria, BP, and HbA1c, the difference in time until death between the high and low risk groups was also not statistically significant (hazard ratio = 1.06, 95% CI 0.73,1.53). Similar results were found as in Table 3 for all baseline covariates along with the time-varying counterparts of albuminuria, BP, and HbA1c. The results verify the findings of Table 3, suggesting that the significant covariates, albuminuria, age, and gender, exert greater effects, compared to DN family history, upon mortality.

Application of a Cox proportional hazards model on the 199 matched pairs (based on propensity score), stratifying by the matched pairs, revealed that risk group still had no significant effect on mortality after reducing the impact of selection (hazard ratio = 1.09, 95% CI = 0.48, 2.47). We excluded three subjects from our survival analyses that died during the study, but no date of death was recorded. Conclusions were unchanged when logistic regression was performed to include these three additional subjects, treating all-cause mortality as binary.

## Discussion

In contrast to some large, longitudinal trials of CKD, which exclude subjects with stage 1 or 2 disease [21,22], our cohort was enriched for patients with preserved GFR, which enabled identification of progression risk factors that might be more amenable to therapeutic intervention. The diabetic subjects with low risk group characteristics (diabetes >10 years, absence of albuminuria and family history of ESRD) did not progress to ESRD during the course of the study. After covariate adjustment by multiple methods, we found that high risk subjects developed faster eGFR decline compared to low risk subjects, implying that DN family history regulates DN progression. GFR decline is customarily depicted to occur 15-25 years after diabetes onset, and following the appearance of overt proteinuria [23]. Although macroalbuminuria was identified as an independent risk for DN progression in our study, eGFR decline was noted when most patients were micro- or normoalbuminuric, and often less than 10 years after diabetes onset.

Several studies have identified family history as a risk for DN progression [24,25], or ESRD due to DN [26]. A unique feature of our study is that history of DN in a sibling predicted early GFR decline, when GFR is relatively preserved, and therapeutic benefit is more likely. The independent effect of family history on eGFR decline suggests that genetic variants regulate DN progression. Intensive efforts are ongoing to identify the multiple, relevant genetic variants for DN [27], and it remains to be determined whether the same genes affect early DN, characterized by microalbuminuria, eGFR changes in the upper register, and modest histological alterations, versus late DN progression, which is characterized by nephrotic range proteinuria, more profound GFR decline, and significant glomerular and interstitial fibrosis. Our dataset is one of the few that could serve as a validation cohort for early DN in type 2 diabetes.

We anticipated that DN family history might also correlate with death, and although there was an association with unadjusted mortality, this hypothesis was not supported by multiple statistical approaches that adjusted for covariates. Because of the divergent correlations between DN progression and mortality with family history, we speculate that different genetic variants may control these two phenotypes. The lack of association between DN family history and mortality is consistent with work by Freedman et al, which showed that diabetic dialysis patients with a family history of ESRD are not more likely to die than those without a family history [28].

Decreased GFR is associated with both ESRD and pre-ESRD mortality, though most studies have not focused on subjects with DN [6-8]. In diabetic patients, the risk for competing ESRD and mortality outcomes depends on study population characteristics, most notably baseline GFR. Several recent reports in subjects with macroalbuminuria and varied GFR at enrollment concluded that ESRD is relatively more common [29-31]. These data are in contrast to UKPDS and Joslin Clinic studies, which demonstrated greater incidence of death compared to ESRD in patients with type 2 diabetes [32,33]. Our cohort experienced 21% mortality over the course of the study, and we observed a greater likelihood of death than progression to ESRD, and a trend toward faster eGFR decline in subjects who died.

One caveat to the earlier trials demonstrating greater relative mortality is that patients were enrolled in an era when advanced cardiovascular interventions and pharmacologic agents, e.g., ACE inhibitors and ARBs, were not routinely administered. However, in our observational cohort, two-thirds of subjects received ACE inhibitors or ARBs, reflecting contemporary standards of care. Furthermore, the mean rate of eGFR decline for the entire study population was 1.5 ml/min/1.73 m^2^/yr, and only 2.2 ml/min/1.73 m^2^/yr in the high risk group, which is lower than other DN trials [9].

One limitation of this study is that GFR was not directly measured. Although iothalamate clearances are considered to be the standard, assay variability is problematic [34], and values weakly associate with CKD complications [35]. More importantly, measured GFR is impractical for large, longitudinal trials. We therefore opted for eGFR calculations, which accurately predict clinical outcomes [8]. Because GFR estimating equations were generated from subjects with renal dysfunction, MDRD- or CKD-EPI-derived eGFR values tend to underestimate GFR in the >60 ml/min/1.37 m^2^ register in cross-sectional analyses. However, this bias is less with longitudinal measurements, and a recent large trial concluded that eGFR change over time is a reliable measure of CKD progression [36]. To enhance robustness of the analyses, we compared eGRF_creat_, eGRF_creat+cysC_ and eGFR_cysC_ between risk groups. The data showed differences between groups, most reliably with eGRF_creat_, to a lesser extent with eGRF_creat+cysC_, and not with the eGFR_cysC_ equation. One explanation for this discrepancy is that our cohort had fewer samples assayed for cystatin C than creatinine. Furthermore, analyses using eGRF_creat+cysC_ required imputation of missing values, which may weaken the correlation [37].

Other potential weaknesses of the study design include discrete entry criteria for high and low risk groups, which may limit conclusions to patients with similar characteristics, rather than the general population. Propensity score matching has inherent limitations, such as the choice of finite co-variates, which creates the possibility that relevant co-variates could be omitted. However, we included the major co-variates associated with most DN studies, and the results of our sensitivity analyses revealed that results were unlikely to be attributed to co-variates with small effect sizes. The propensity matched samples represented a subset of the entire population, so the smaller sample size may therefore reduce power. Annual measurements for systolic BP, diastolic BP, HbA1c and urine albumin:creatinine ratios were collected. However, these time-varying covariates have the potential for measurement error and a false attribution to cause, if the predictor variable is associated with the error term. We note these potential limitations, which we address using the counting process style of input, when evaluating our extended Cox model with these time-varying covariates. However, because of these potential biases, we could not incorporate these covariates into our mixed effects models. Instead, we are applying joint model methodology to examine the longitudinally measured covariates and eGFR (DG, AS, and JS, manuscript in preparation).

## Conclusions

In our diabetic cohort with mean eGFR = 74 ml/min/1.73 m^2^ albuminuria and family history of DN are significant risks for GFR decline and progression to ESRD, whereas subjects with diabetes for longer than 10 years, normoalbuminuria and negative family history, experience slower eGFR decline, and are extremely unlikely to require dialysis. Although we would not recommend that patients with low risk characteristics be neglected, scarce resources would be more sensibly devoted to vulnerable patients, such as the high risk cases in our study, and preferably prior to the onset of albuminuria or GFR decline.

## Abbreviations

DN: Diabetic nephropathy; eGFR: Estimated glomerular filtration rate; ESRD: End stage renal disease; HbA1c: Hemoglobin A1c.

## Competing interests

The authors declare no competing interests.

## Authors’ contributions

AJB, JRSch, JRSe and SKI designed the study. DG and JRSch drafted the manuscript. AJB, AO, AS, JRSe and SKI critiqued and revised the manuscript. AO and RLT acquired the data and processed specimens. AJB, JRSch, JRSe and SKI obtained funding. AS, CT, DG and GBR performed the statistical analysis. All authors read and approved the final manuscript.

## Pre-publication history

The pre-publication history for this paper can be accessed here:

http://www.biomedcentral.com/1471-2369/14/124/prepub

## Supplementary Material

Additional file 1: Figure S1 Creatinine assay validation from 40 samples. A, original study creatinine value (X-axis) versus quality control creatinine assay value obtained at Cleveland Clinic Foundation (CCF) reference laboratory (Y-axis). The two values were highly significantly correlated, with a mean difference between measurements = 0.07 mg/dl. B, Bland-Altman plot measuring the difference between the two assay values for each sample (Y-axis) versus mean values between samples (X-axis). **Table S1**. Comparison of baseline patient characteristics between centers. **Table S2**. Baseline covariate effects on eGFRcreat+cysC using linear mixed effects model parameter estimates, 95% confidence intervals and p-values. Click here for file
