# Supplementary material for: Diabetic nephropathy in a sibling and albuminuria predict early GFR decline: a prospective cohort study
Source: BMC Nephrol. 2013 Jun 17;14:124. doi: 10.1186/1471-2369-14-124 (PMC3703258; doi:10.1186/1471-2369-14-124)
Supplement: Additional file 1: Figure S1 — Creatinine assay validation from 40 samples. A, original study creatinine value (X-axis) versus quality control creatinine assay value obtained at Cleveland Clinic Foundation (CCF) reference laboratory (Y-axis). The two values were highly significantly correlated, with a mean difference between measurements = 0.07 mg/dl. B, Bland-Altman plot measuring the difference between the two assay values for each sample (Y-axis) versus mean values between samples (X-axis). Table S1. Comparison of baseline patient characteristics between centers. Table S2. Baseline covariate effects on eGFRcreat+cysC using linear mixed effects model parameter estimates, 95% confidence intervals and p-values. [file 1471-2369-14-124-S1.pdf]

Figure 1

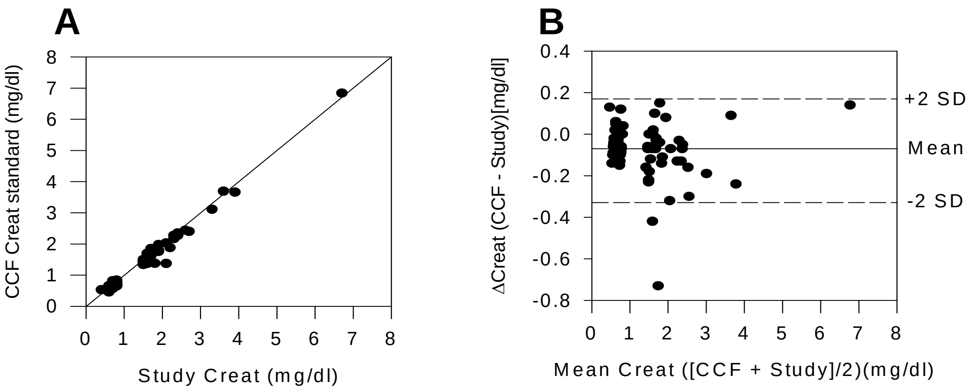

Creatinine assay validation from 40 samples. A, original study creatinine value (X-axis) versus quality control creatinine assay value obtained at Cleveland Clinic Foundation (CCF) reference laboratory (Y-axis). The two values were highly significantly correlated, with a mean difference between measurements = 0.07 mg/dl. B, Bland-Altman plot measuring the difference between the two assay values for each sample (Y-axis) versus mean values between samples (X-axis).

Table 1. Comparison of baseline patient characteristics between centers

	Cases (CWRU)	Cases (WFU)	p-value	Controls (CWRU)	Controls (WFU)	p-value
N	298	137		280	120	
Gender (% female)	64.8	58.4	0.20	72.5	54.2	< 0.01
Age (yr)	58.7 ± 10.7	59.2 ± 10.2	0.65	57.9 ± 12.0	64.6 ± 9.8	< 0.01
Race (% AA)	51.0	52.6	0.76	48.6	61.7	0.02
Diabetes duration (yr)	14.6 ± 9.5	12.9 ± 9.9	0.09	17.2 ± 9.2	17.2 ± 7.8	1.00
HbA1c (%)	7.7 ± 1.9	7.6 ± 1.8	0.60	7.9 ± 2.1	7.5 ± 1.5	0.06
Systolic BP (mm Hg)	136.2 ± 18.9	133.6 ± 15.5	0.16	134.2 ± 21.8	134.2 ± 15.8	1.0
Diastolic BP (mm Hg)	75.8 ± 12.3	76.0 ± 10.3	0.87	74.6 ± 13.2	74.8 ± 9.6	0.88
$eGFR_{creat}$ (ml/min/1.73 m^2)	73.5 ± 29.0	76.0 ± 24.5	0.38	77.5 ± 22.1	72.9 ± 20.1	0.05
$eGFR_{creat+cysC}$ (ml/min/1.73 m^2)	73.9 ± 30.8	74.0 ± 25.7	0.97	78.6 ± 24.6	74.7 ± 22.8	0.14
Urine alb:creat (mg/g)	33 (10-139)	19 (8-87)	0.36	12 (7-22)	9 (5-19)	<0.01

Data are presented as mean ± standard deviation for continuous measures, % frequency of reference level for discrete measures and median (first quartile-third quartile) for urine alb:creat (mg/g). Analyses to generate p-values include t-tests, χ^2 tests and Wilcoxon rank-sum tests where appropriate.

Table 2. Baseline covariate effects on $eGFR_{creat+cysC}$ using linear mixed effects model parameter estimates, 95% confidence intervals and p-values

Effect	Estimate (β_{Basic})	95% CI	p-value
**Risk group (high vs. low)**	1.65	(-1.32, 4.62)	0.277
**Year**	-0.90	(-1.60, -0.21)	0.012
**Risk group by year interaction**	-1.38	(-2.18, -0.58)	<0.001
**Diabetes duration**	0.00	(-0.12, 0.12)	0.955
**$eGFR_{creat+cysC}$**	0.76	(0.71, 0.80)	<0.001
**Urine alb:creat ratio**	-2.94	(-5.04, -0.84)	0.008
**Systolic BP**	-0.05	(-0.11, 0.02)	0.147
**Diastolic BP**	0.05	(-0.06, 0.16)	0.371
**HbA1c**	-0.11	(-0.85, 0.63)	0.774
